# Revealing four decades of snow cover dynamics in the Hindu Kush Himalaya

**DOI:** 10.1038/s41598-022-17575-4

**Published:** 2022-08-04

**Authors:** K. Naegeli, J. Franke, C. Neuhaus, N. Rietze, M. Stengel, X. Wu, S. Wunderle

**Affiliations:** 1 Institute of Geography and Oeschger Center for Climate Change Research, University of Bern, Hallerstrasse 12, 3012 Bern, Switzerland; 2 Department of Geography, University of Zurich, Winterthurerstrasse 190, 8057 Zurich, Switzerland; 3 Deutscher Wetterdienst, Frankfurter Str. 135, 63067 Offenbach am Main, Germany; 4 College of Earth and Environmental Sciences, Lanzhou University, Lanzhou, 730000 China

**Keywords:** Environmental impact, Cryospheric science

## Abstract

Knowledge about the distribution and dynamics of seasonal snow cover (SSC) is of high importance for climate studies, hydrology or hazards assessment. SSC varies considerably across the Hindu Kush Himalaya both in space and time. Previous studies focused on regional investigations or the influence of snow melt on the local hydrological system. Here, we present a systematic assessment of metrics to evaluate SSC dynamics for the entire HKH at regional and basin scale based on AVHRR GAC data at a 0.05° spatial and daily temporal resolution. Our findings are based on a unique four-decade satellite-based time series of snow cover information. We reveal strong variability of SSC at all time scales. We find significantly decreasing SSC trends in individual summer and winter months and a declining tendency from mid-spring to mid-fall, indicating a shift in seasonality. Thanks to this uniquely spatio-temporally resolved long-term data basis, we can particularly highlight the unique temporally variable character of seasonal snow cover and its cross-disciplinary importance for mountain ecosystems and downstream regions.

## Introduction

**Figure 1:**
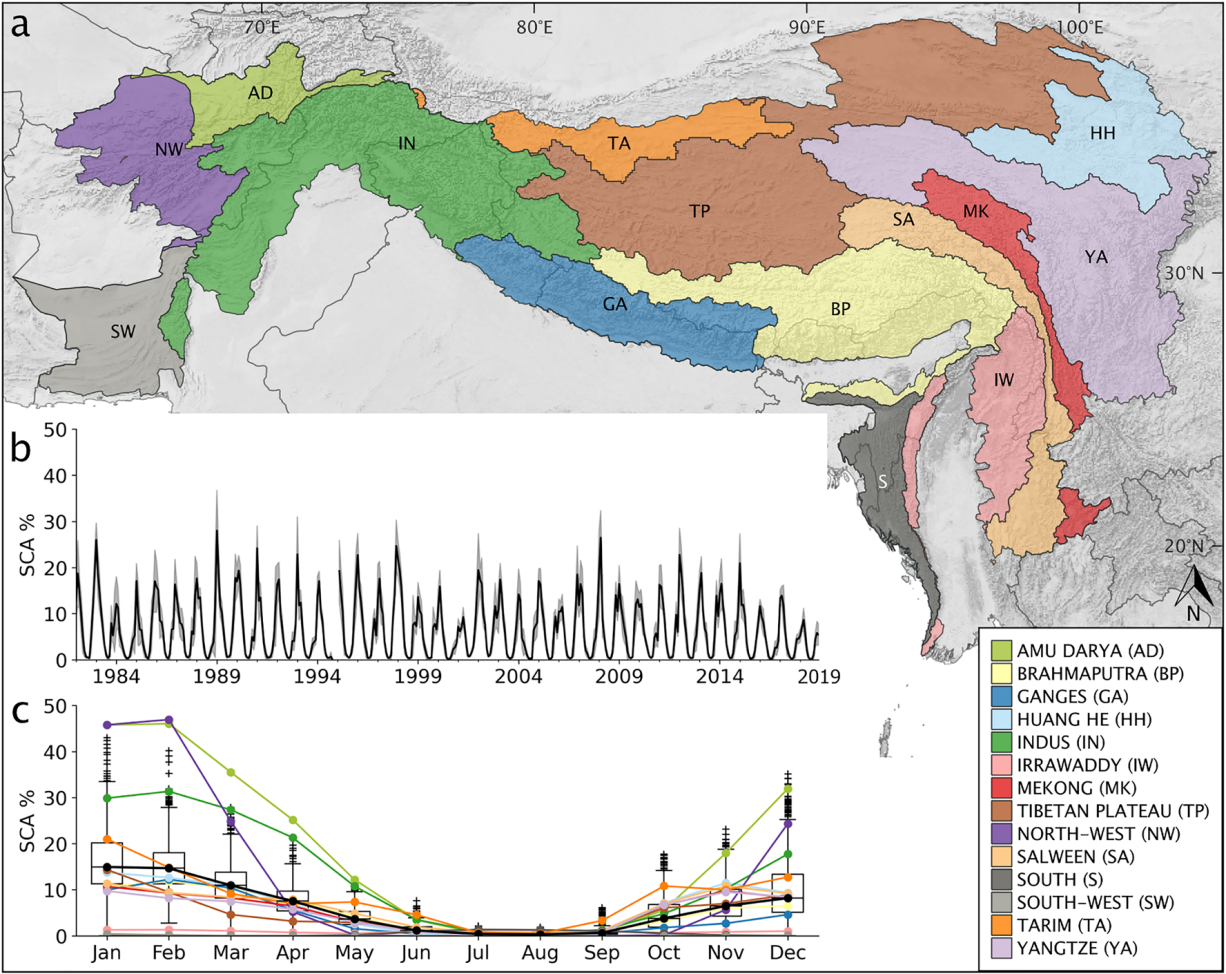
The Hindu Kush Himalaya region and its spatial seasonality of snow cover occurrence. (**a**) The map shows the entire HKH extent and the subdivision into 14 major river basins. Please not some individual basins are cut by the HKH outline applied. (**b**) Snow Cover Area Percentage time series, including monthly standard deviation (grey shaded area), for the HKH and the entire study period 1982–2018. (**c**) Monthly median of SCA % averaged over the entire study period for the whole HKH region (black) and all individual basins (coloured lines) over the course of the year. SCA values are given in numbers relative to the size of the respective region. Boxplots show median and IQR, whiskers the quartile to extreme ranges, and outliers are shown as crosses for the time series of the whole HKH region. All numeric details of c can be found in the Supplementary Table S2.

Two billion people, a fourth of the world’s population, live in the HKH region. It covers ~ 4.2 million km^2^ and extends over eight countries from 61° to 105° East and 16° to 40° North. The basis for livelihoods is affected by changing runoff patterns that provide ecosystem services^1^. Local communities are threatened by cascading events, often interlinked with high snow amounts, that require disaster risk management but also hazard monitoring^2^. Hydrologically, the HKH is the headwater area of Asia’s largest rivers and can be subdivided into ten basins, of which some are only partly captured within this outline (Fig. 1a). Its great size and complex topography make the HKH climatically strongly heterogeneous with regions featuring (sub-)tropical at lower and alpine climate at higher elevations. While the West is influenced by winter westerly disturbances^3,4^, the Indian Summer and East Asian Monsoon are the major moisture sources for the central and eastern Himalaya, respectively^5^. Annual precipitation amounts are thus highly variable ranging from < 500 mm (Indus basin) to > 2000 mm (Brahmaputra basin)^6^. Seasonality of precipitation is varying from being received mainly (80% and more) in summer in the central Himalaya to being more equally distributed with considerable amounts (50% and more) of winter precipitation in the West and North-East^5,7^. Mean winter and summer air temperature range from well-below freezing point to ~ 20 °C and ~ 0° to well-above 30 °C, respectively^8^. Cryospheric components strongly contribute to the freshwater resource within HKH^9^. While their share to runoff in the East plays a minor role^10^, ice and snow melt makes up to > 60% of the annual discharge in the West (Amu Darya, Indus basin), particularly in the upper basins and during dry seasons^5,11–13^. This is of particular importance for water-scarce regions in the lowlands that are densely populated and highly dependent on regular stream flow^9^.

Here, we used seasonal snow cover (SSC) information derived from seven Advanced Very High Resolution Radiometer (AVHRR) sensors onboard NOAA-satellites, produced within the frame of the ESA Climate Change Initiative Snow project^14^. This unique dataset of exceptional temporal length and frequency enables to tackle the spatio-temporal long-term (1982–2018) dynamics of SSC. The snow cover information is retrieved from a Fundamental Climate Data Record (FCDR)^15^ providing multi-spectral, medium resolution daily global composites of reflectance and brightness temperature data. The timeseries is intercalibrated and geolocated to ensure long-term stability^16^. A semi-empirical method^17,18^ was chosen to obtain daily snow maps. A temporal gap-filling reduced the inference of clouds^19^. SSC metrics, snow cover area percentage (SCA%) and snow cover duration (SCD), at varying spatio-temporal scales are derived (cf. “Materials and methods”).

The SSC time series shows great accordance with snow cover presence at in situ stations (overall accuracy of 94%, based on 118 in situ stations between 1982 and 2013) and high-resolution satellite imagery (correlation coefficient of 0.82, based on 201 Landsat scenes between 1999 and 2018)^20^. Temporal consistence of the SSC time series is proven by the comparison with two independent datasets, the fractional snow cover from Modern-Era Retrospective analysis for Research and Applications version 2 (MERRA2)^21,22^ and the NOAA Climate Data Record (CDR)^23,24^ (cf. “Materials and methods”, Supplementary Fig. S1).

## Results and discussions

### Spatio-temporal variability of seasonal snow cover extent

SSC in the HKH has a distinct seasonality with greatest extent in winter and spring months and being least in summer (Fig. 1c). Snow melting season can be identified between March and June, followed by an almost snow-free summer (July and August) period. During fall (September to October) the snow cover gradually evolves with a sharp increase at the beginning of winter (cf. Supplementary Table S2). Looking at the monthly SSC over the entire time series, the years 1983, 1989 and 2008 show maximum and the years 2001, 2016 and 2018 minimum extents, respectively (Fig. 1b). At the basin scale, the Amu Darya and the North-West basins exhibits SCA extent of over 50% in January and February (Fig. 1c). The other mountainous western basin Indus also shows SCA values of around 30% in high winter. Applying the HKH outline cuts some basins, in particular Tarim, Amu Darya and North West, which likely exhibit stronger SCA extents not captured within this study. Due its great size and the predominant winter westerlies precipitation, the Indus basin most strongly dominates the HKH-wide SSC dynamics. All other basins are clearly less affected by SSC, but not the basins Irrawaddy, South and Southwest where SSC almost never occurs. Interestingly, the Tibetan Plateau shows the flattest seasonal distribution of SSC, mainly governed by low winter precipitation and the high occurrence of ephemeral snow^25^. Throughout the year, SSC in most basins follows the general seasonality, except for the Irrawaddy and South basins that although affected little by SSC undergo an inverse yearly course with maximum SSC in July and August. This opposite pattern is an interplay of elevation and the Indian summer monsoon, the main precipitation source for this region^5^.

**Figure 2:**
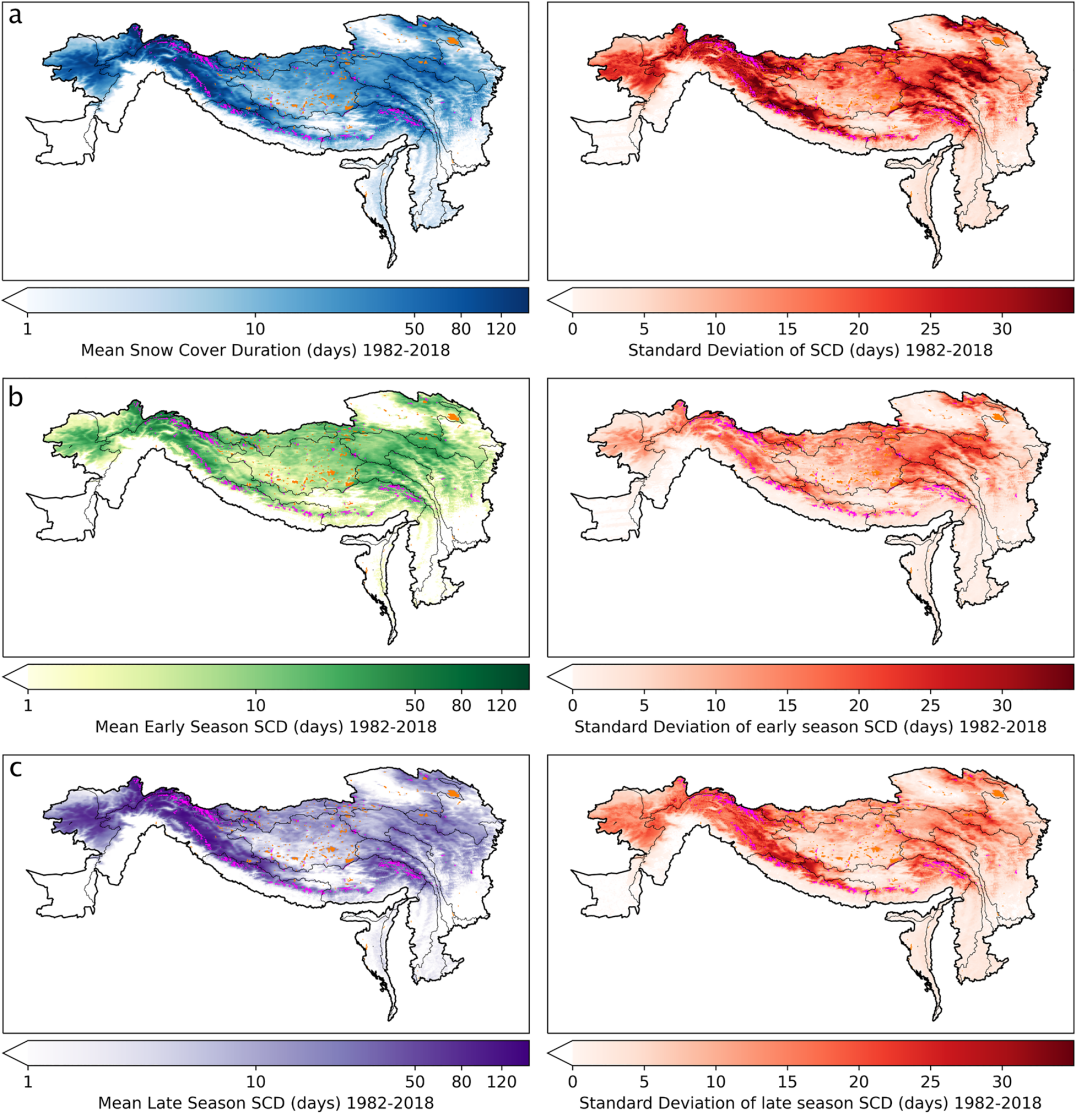
Seasonal Snow Cover regime and its variability over time (**a**) Mean snow cover duration (left). (**b**) Mean early season snow cover duration, SCD_es_ (left). (**c**) Mean late season snow cover duration, SCD_ls_ (left). Standard deviations of mean SCD, SCD_es_ and SCD_ls_ are given in the right column. All displays present averaged data between 1982 and 2018. Early season is defined as between 1 September and 15 January of a given hydrological year, while late season is defined as between 16 January and 31 August. Permanent ice is displayed in pink, masked water bodies in orange. Geolocation and scale according to Fig. 1.

The SCD varies strongly over the HKH region (Fig. 2a). The longest mean SCD is observed in the mountainous Amu Darya and Indus basins (at maximum up to 339 days). Other mountain ranges, like the Nyainqentanglha, exhibit SCD of up to ~ 120 days on average and ~ 230 at maximum. Although situated at high elevation, the Tibetan Plateau is covered by snow in < 50 days per year on average and up to ~ 140 days at maximum (cf. season SCD^26^) and are supporting the pattern found in mean SCD. Areas with extended SCD also exhibit earlier snow onset and later melt-out, respectively. The standard deviations (STD) plots (Fig. 2 right column) indicate regions with greatest and least variability in SCD (overall, early, late) within the period 1982 to 2018. While lower elevated regions as well as the Tibetan Plateau show generally high stability in SCD, mountain ranges are most affected by variable SCD. Greater variability is found for the late season compared to the early season indicating changes in the melt out period of the snow cover extent. As previous studies are mainly based on Moderate Resolution Imaging Spectroradiometer (MODIS) data and thus cover only half of our study period (at maximum 2001 to today, e.g.^27–29^), we here also investigate the long-term change in variability. Therefore, the mean STD for the pre-MODIS era (1982–2000) is subtracted from the mean STD of the MODIS era (2001–2018, cf. Supplementary Fig. S3). Thus, regions marked by positive (negative) ΔSTD exhibit an increase (decrease) in variability in the more recent two decades. While large areas did not undergo a change in variability, the Tibetan Plateau and Tarim basins experienced a decrease in variability over time (brownish areas). An increase in variability is only patchy distributed over the main Himalayan mountain range and in the north-eastern corner of HKH bording China (Danghe Nanshan, Tergun Daba Shan, Datong Shan). SCD_es_ mainly resembles these variability changes, while SCD_ls_ generally exhibits less variability over time.

### Trends in snow cover extent

**Figure 3:**
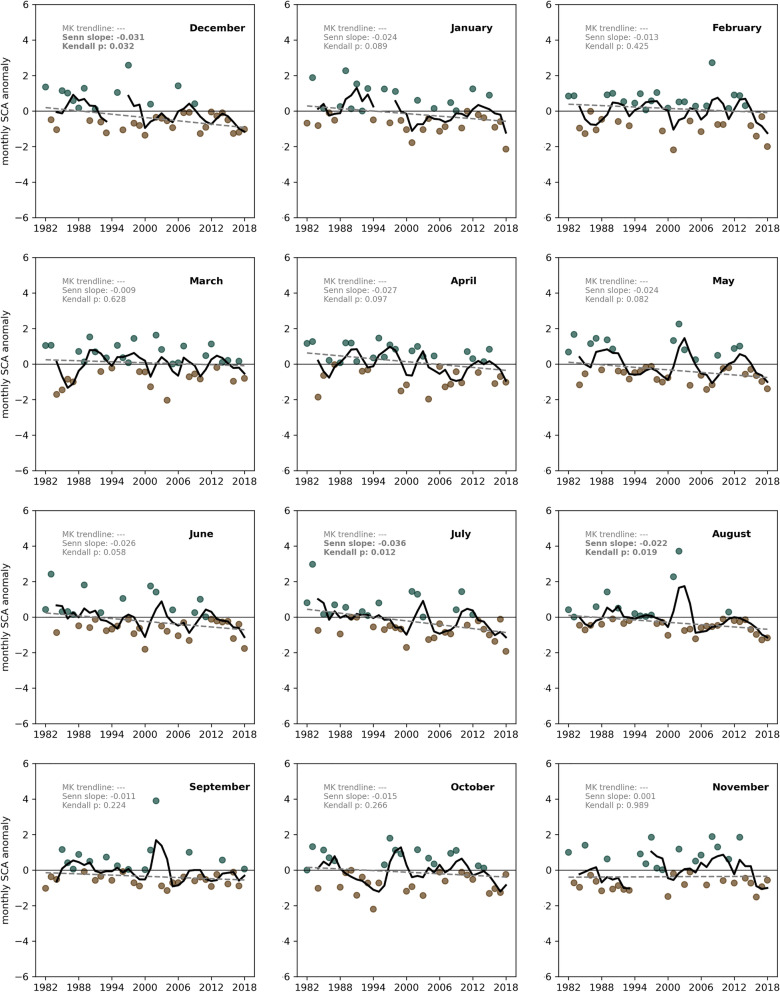
Seasonal changes in snow cover extent. Monthly SCA anomalies for the entire HKH region with respect to the 1982–2010 reference period. Teal and brown dots denote positive and negative yearly anomalies, respectively. Solid black lines denote 3-year running mean, dashed lines denote the MK trendline and statistics (Senn slope and Kendall p) are given at the top of each box (bold indicates significant values). Missing data from November 1994 to February 1995 are visible. All numeric details can be found in the Supplementary Table S3.

Variations in SSC phenology are a direct response to climate change^30^, mainly temperature and precipitation, and thus of high relevance for seasonal water storage and mountain streamflow^9^. Changing snow cover duration have far-reaching consequences^31,32^. We see a negative trend in most months, however not always significant yet, because of the high interannual variability (Fig. 3). Strongest decline in SCA percentage is recorded between April to August as well as December and January (Senn slope − 0.027 on average), with significant negative trends in December, July and August. All other months when SSC is either slowly diminishing or building up, show greatest interannual variability impeding a clear long-term trend. Seasonal long-term investigations revealed a similar picture, i.e. significant negative trends for ‘winter’ (DJF; Senn slope − 0.04) and ‘summer’ (JJA; Senn slope − 0.03), and a negative yet not significant trend for ‘spring (MAM) and ‘fall’ (SON) (cf. Supplementary Fig. S6).

**Figure 4:**
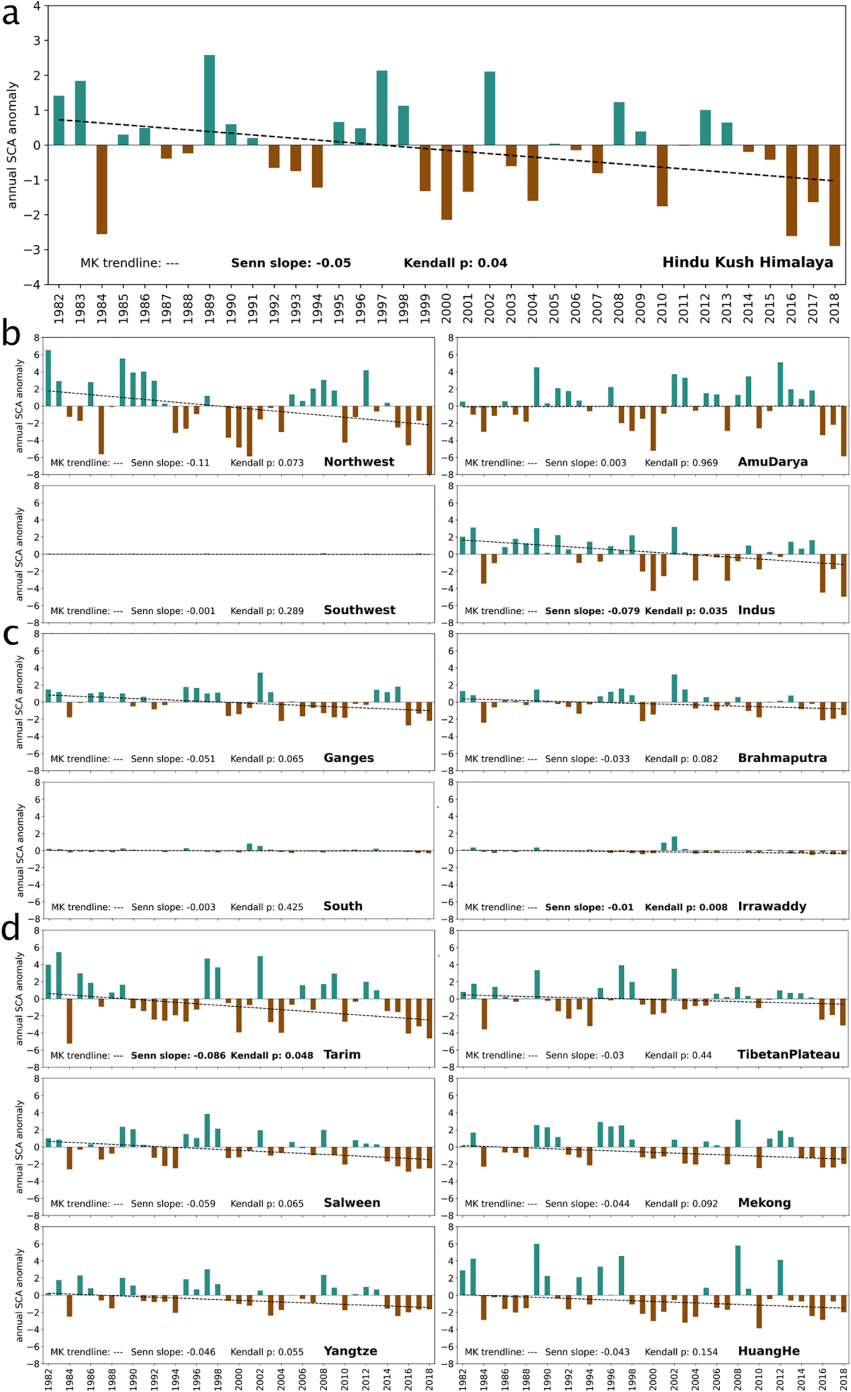
Interannual and spatial variability in snow cover extent. Annual SCA % anomalies for the entire HKH region (**a**), or one of the 14 basins; arranged in greater regions west (**b**), central (**c**) and east (**d**), with respect to the 1982–2010 reference period. Note different y-axis range for the top HKH region figure. Dashed lines denote the MK trendline and statistics (Senn slope and Kendall p) are given at the bottom of each graph. All numeric details can be found in the Supplementary Table S4.

The interannual variability of SSC in the HKH is also strikingly high at the annual resolution but with a significant negative trend (Senn slope − 0.05, Fig. 4a). At the basins scale, all but Amu Darya experience negative trends, although only significant for the Indus and Tarim basins (Fig. 4b–d). For most of the basins and the entire HKH region, the years 1984, 2000 and 2016 to 2018 were strongly negative. Besides these years, two periods around the years 1993 and 2000 are characterised by consecutive negative anomalies. In contrast, around years 1990, 1997 and 2012 short periods of increased SSC occurrence can be observed in the HKH and many individual basins. Basin-wide trends should be treated with caution as some extents are cut by the HKH outline applied.

**Figure 5:**
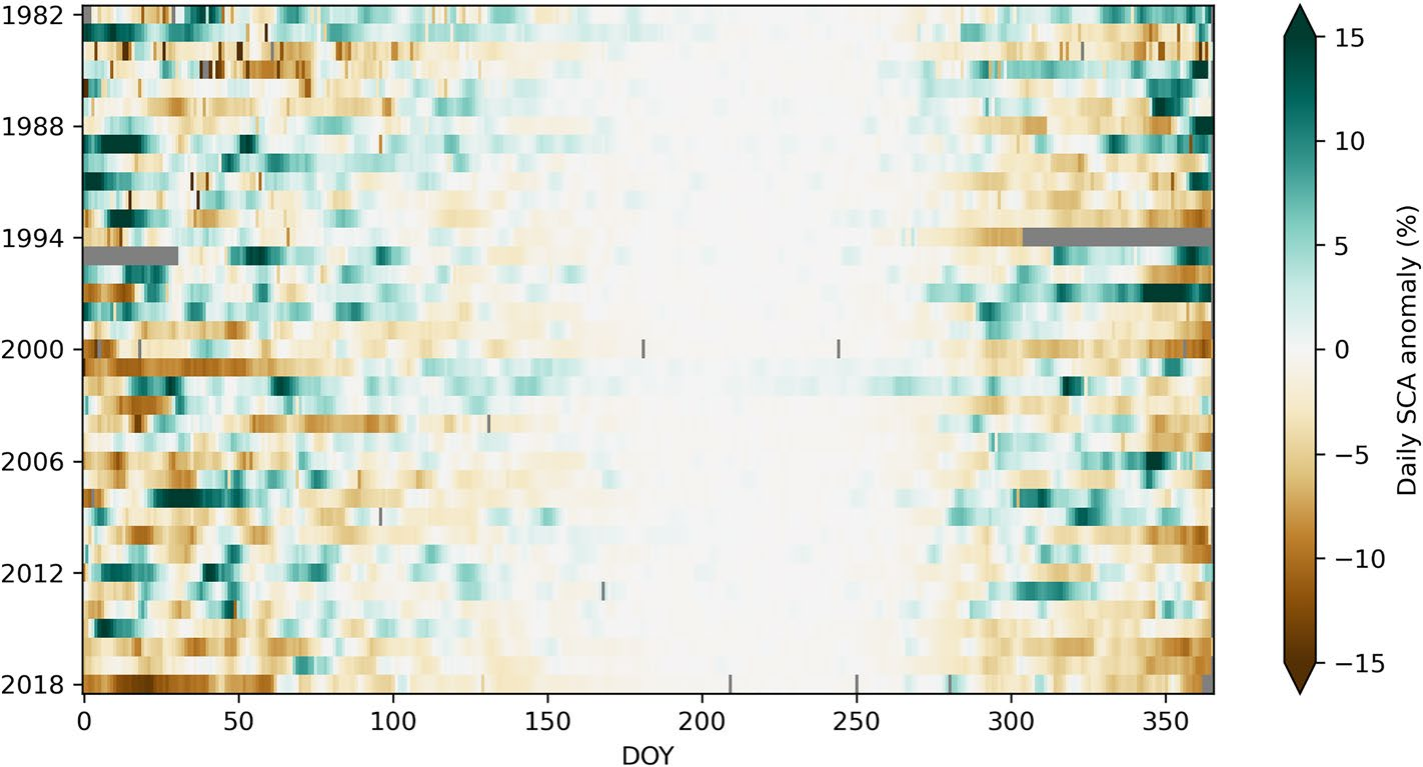
Long-term seasonal snow cover variability in the HKH. Heat map of daily SCA anomalies over the study period 1982–2018 with respect to the 1982–2010 reference period. Missing data from November 1994 to February 1995, and some individual days, are marked in grey.

To evaluate long-term changes in SSC in detail, we investigated SCA percentage anomalies at the highest available temporal frequency. Figure 5 depicts this information averaged for the HKH and resolved over the full study period at daily resolution. Yet at this temporal resolution SSC is highly variable. The enlarging bright-coloured area in more recent years (DOY 75-280) supports our findings of a shortening of SSC occurrence (cf. Fig. 3). In particular, between DOY 75 and 160 the anomalies are closer to zero in more recent years pointing towards an earlier snow melt-out. Both negative and positive extremes (e.g. winter 2001 and 2018 or winter/spring 1997 and 2012, respectively) are readily apparent, highlighting the great temporal variability of SSC in the HKH region. These rapid anomaly changes emphasise the unique temporally variable character of snow cover dynamics compared to other cryospheric components.

### Comparison with other regional snow cover extent estimates

As of today, no study has investigated seasonal snow cover metrics for the entire HKH region over such a long time (four decades) at medium spatial (0.05°) and with high temporal (daily) resolution. Studies focused on regional assessments and/or limited time periods, or continental scale assessments at coarser spatial resolution, leading to inconsistent and weak findings^9^. Thus, a comparison with other studies is not directly feasible due to the different temporal and spatial coverage, not to forget the different methods applied and data sets used.

Nevertheless, we would like to stress that our findings align well with recent studies examining snow cover dynamics over continental to hemispherical scales but over much shorter timescales^29,30,33–35^. In particular, the observed high interannual variability often impeding clear long-term trends is also observed by smaller-scale regional studies, and mainly linked to elevation, monsoon-influence and heterogeneously changing temperature and precipitation patterns^28,36–38^. The detected shift in seasonality and extended snow-free period caused by an earlier melt-out is confirmed by multiple studies^4,25,34,39–41^ based on varying datasets and focusing at different spatio-temporal scales. Moreover, findings of recent studies looking at spatio-temporal distribution^42^ and long-term trends^43^ of Snow Water Equivalent (SWE) in this region, agree well with our overall findings of great heterogeneity and variability.

At the basin scale, our findings deliver unique long-term insights that reflect local characteristics. Western basins (Fig. 4b) that are known to be provided up to > 60% of the annual discharge by snow melt^5,11–13^ and mountainous basins such as Tarim are clearly affected by the greatest magnitude in interannual variability in SSC. However, while the Indus, Northwest and Tarim basins exhibit negative trends in SCA % long-term evolution, the strong interannual variability in the Amu Darya impedes the trend formation.

### Possible drivers of seasonal snow cover dynamics

The presence of snow is strongly linked to air temperature and precipitation. However, the interplay is complex and lively. While temperatures above zero degrees Celsius prevent solid precipitation, the ground can still be covered by snow while it is raining. Nevertheless, temperature and precipitation data provide an insight to local situations and hint explanations for extreme situations. To track down some of the snow cover variability in the HKH, we assessed the interplay of SSC with temperature and precipitation using CRU TS v4.0 and CHIRPS data, respectively (cf. “Materials and methods”). Both temperature and precipitation show clear positive trends at regional HKH and basin scale with most of these trends being significant (Supplementary Figs. S3 and S4). Negative snow cover anomalies usually occur alongside negative precipitation anomalies, whereas positive snow cover anomalies require mainly colder temperatures (cf. e.g. winters 1999 to 2006 and 1989 to 1993 or 1998 in the Supplementary Fig. S5, respectively).

Seasonal means of air temperature and precipitation impede the assessment of short-term variability but are suitable to explain extremes. However, due to the rather sluggish behaviour of SSC compared to temperature and precipitation, a previous season might influence the following. Thus, extensive SSC in summer is likely strongly influenced by the preceding spring and winter SSC distribution and not only driven by summer temperature and precipitation patterns. To be able to provide in-depth results of this complex interplay further data would be needed (e.g. automatic weather station data).

### Present and future implications of spatio-temporal occurrence of SSC

The detected high variability of SSC at all spatio-temporal scales poses major challenges to local communities, requires a new level of preparedness and calls for adaptation strategies. An elevation dependent warming over the Tibetan Plateau^44^, increased warming in mountains^8,45^ and seasonally pronounced warming in winter^46^ are known to be major drivers of changing SSC occurrence due to shifting rain-snow precipitation ratios^12^. Earlier spring discharge peaks lead to water shortage in dryer summer months affecting communities living along and depending on the major rivers originating in the highest elevations of the HKH. It is important to stress the far-reaching consequences of changing SSC. Changes in vegetation distribution^47,48^, runoff patterns^5^ and thus agricultural productivity^49^ are highly dependent on snow and ice melt and thus closely linked to changing SSC. Furthermore, the HKH is particularly prone to cascading events triggered by a variety of individual hazards such as floods, avalanches, landslides or droughts^50^. SSC extremes are likely to intensify such natural hazards at local to regional scale.

HKH-wide projections for the second half of the twenty-first century are still uncertain^51^, but announce an elevation-dependent decrease with a sustained strong interannual variability in SSC^45^ leading to a shift towards earlier snow melt and thus increased runoff in late spring and early summer (May and June)^52^. Basins with major precipitation input in winter (e.g. Indus basin) will be more affected by a changing snow-rain ratio and suffer a greater overall loss in SSC due to the non-uniform behaviour of temperature, precipitation and snow fall. This will reduce water availability and thus threaten food security in most of the HKH, particularly the Indus and Brahmaputra basins that are highly vulnerable to water scarcity^13,31^.

In this study, we provide a comprehensive assessment of SSC dynamics in the HKH. For the first time thanks to the unique spatio-temporally resolved data basis over multiple climate periods the high variability of SSC indicating a complex interplay between regional climate, continental warming trends, and influence of circulation pattern for precipitation events is revealed. Our independent satellite-based long-term SSC product can serve as highly valuable input data to improve predictive modelling assessments at climate-relevant timescales. Moreover, this dataset provides the basis for cross-scale transdisciplinary studies. Better understanding of the current variability and trends in SSC will improve climate resilience in this densely populated and rapidly changing environment.

## Materials and methods

### Dataset

In this study, we used daily, global snow cover extent information (https://doi.org/10.5285/3f034f4a08854eb59d58e1fa92d207b6)^14^ processed in the frame of the ESA CCI + Snow project and based on the Advanced Very High Resolution Radiometer (AVHRR) Global Area Coverage (GAC) Fundamental Climate Data Record (FCDR) (https://doi.org/10.5676/DWD/ESA_Cloud_cci/AVHRR-PM/V003) produced in the ESA Cloud CCI project^15^ using PyGAC^16^. The unique FCDR encompasses a time span of exceptional length (1982 to 2016) at a daily temporal resolution and was extended using the same pre-processing and retrieval algorithm for years 2017 and 2018 for the ESA CCI + Snow project. The composites cover the entire globe (− 180°, 90°, 180°, − 90°) at a spatial resolution of 0.05 × 0.05° pixel size. The data originates from both AVHRR-2 and AVHRR-3 sensors on-board NOAA-07, -09, -11, -14, -16, -18 and -19 satellites. AVHRR is a multi-spectral sensor with two (three if channel 3a is available) channels in the visible/infrared spectral range and two (three) channels in the thermal spectral range (see Supplementary Table S5). Due to sensor problems some data gaps do exist in the data record, mainly November 1994 to January 1995 and some individual days. Finally, our gap-filled AVHRR FCDR comprises two longer data gaps of 92 and 21 days respectively, 16 individual daily gaps and each 3 days missing information at the beginning and end of the time series due to the temporal gap filling, resulting in 99% data coverage over the entire study period of 37 years.

### Auxiliary datasets

The detection of clouds in passive VIS/IR satellite data is crucial to optimally derive snow cover extent data. Here, we used the cloud mask, including a uncertainty estimation mask, provided by ESA Cloud CCI^15^. Pixels are marked as cloudy if the cloud mask is positive (equal to 1) and the uncertainty estimate is low (uncertainty ≤ 20%). Land cover information, in particular a water mask and a permanent ice mask, were obtained from ESA Landcover CCI for the year 2000^53,54^. Pixels that are partly affected by water or ice/snow are masked out if more than 50% are covered by water or ice/snow, respectively. While the water mask covers about 1%, the permanent ice mask affects about 1.3% of the entire HKH region. Moreover, polar night is assumed if the solar zenith angle exceeded 88°, which is for the HKH region very rarely applicable. The information about water, permanent ice as well as polar night is coded per pixel in the final snow cover fraction product^14^.

To retrieve snow cover on ground we use a forest canopy transmissivity map that is based on the tree cover classes of the ESA CCI Land Cover 2000 data and the tree cover density map from Landsat data for the year 2000^55^. This transmissivity layer is used to apply a forest canopy correction and thus enables to estimate the fractional snow cover on ground in forested areas.

We used the hydrological basin boundaries provided by the GRDC Major River Basins of the World (MRB) GIS project of the Global Runoff Data Centre (GRDC)^56^. Please note that some basins are cut by the HKH outline and thus are not fully captured in this study.

Near-surface air temperature data is used from CRU TS (Climatic Research Unit gridded Time Series) version 4.0 (http://doi.org/10/gbr3nj) that provides monthly temperature data based on local weather stations available from 1901 to present including an estimation of the data quality at a 0.5° gridded resolution^57^. This dataset has been widely used in the HKH region and correlations with station data at varying elevations are high^46^.

Precipitation data is used from the CHIRPS (Climate Hazards group Infrared Precipitation with Stations) dataset, which retrieves precipitation information based on thermal and infrared satellite data blended with gauging station data at a quasi-global 0.05° gridded resolution from 1981 to present^58^. Over the HKH region, precipitation datasets that include gauging station information tend to be most accurate^59^ and thus CHIRPS with its long time period covered is ideal to be used in this study.

The temporal consistency of our SSC timeseries is proven by the comparison to the fractional snow cover data from Modern-Era Retrospective analysis for Research and Applications version 2 (MERRA2)^21,22^ and the NOAA Climate Data Record (CDR) snow cover product, see Supplementary Fig. S1^23,24^. MERRA2 is a long-term (1980 to today) coarse resolution (50 km) global assimilation reanalysis product. It provides snow water equivalent from which snow cover fraction can be derived. The dataset and its performance has been compared to other snow cover products at great depth^51,60^. NOAA CDR is a long-term (1967 to today) coarse resolution (190 km) multi-source satellite snow cover product covering the Northern Hemisphere. The dataset is not interfered by clouds due to weekly compositing prior to June 1999 and the inclusion of passive microwave data posterior. The NOAA CDR has been widely used in climate-snow studies mostly at hemispherical scale^61,62^.

### Fractional snow cover retrieval

The retrieval of fractional snow cover is a multi-step approach including a pre-processing, the retrieval algorithm based on a feasible method to map Snow Cover Area also in forested regions (SCAmod^17,18^) and a post-processing. Physically the approach makes use of the high reflectance of snow in the visible spectra compared to other natural targets and is thus mainly based on the visible red channel (AVHRR channel 1 centred around 0.63 μm).

The pre-processing identifies all cloud-free pixels which are certainly snow-free based on the Normalised Difference Snow Index (NDSI), which makes use of the spectral bands centred at around 0.63 μm (AVHRR channel 1, ${vis}_{red}$) and 1.61 μm (AVHRR channel 3a or the reflective part of channel 3b, $SWIR$), and an emissive band centred at about 10.8 μm (AVHRR channel 4, ${BT}_{4}$). The reflective part of channel 3b is derived after^63^:

1 $$ref3b= \frac{{I}_{3}(measured) - {I}_{3}\left(B{T}_{4}\right)}{\frac{1}{\pi }\left({F}_{3}{\mu }_{0}\right) - {I}_{3}(B{T}_{4})}$$

where ${I}_{3}\left(measured\right)$ is the measured radiance (mW m^−2^ sr^−1^ cm) for channel 3b, ${F}_{3}$ is the integrated solar spectral irradiance (mW m^−2^ cm) weighted by the spectral response function for channel 3b, and ${\mu }_{0}$ is the cosine of the solar zenith angle. To account for changing solar zenith (sunz) angles the Top of Atmosphere (TOA) reflectance was corrected by a division of the cosine(sunz). This results in a certainly snow-free pixel mask, ${px}_{snow-free}$, that is applied to all data:

2 $${px}_{snow-free} \, if \, vis_{red}<0.12 \, OR \, {BT}_{4}>283^\circ \mathrm{K } \, OR \, {NDSI}_{fsc}<5\%$$

where the fractional snow cover information ${NDSI}_{fsc}$ is obtained by applying the linear relationship after^64,65^:

3 $${NDSI}_{fsc}= -0.01+1.45*NDSI$$

with $NDSI$ being the Normalised Difference Snow Index:

4 $$NDSI= \frac{vis_{red}-SWIR}{vis_{red}+SWIR}$$

The core of the retrieval algorithm is the SCAmod model defined as:

5 $${SCA}_{mod}= \frac{\frac{1}{{t}_{\lambda }^{2}} * {vis}_{red} + \left(1 - \frac{1}{{t}_{\lambda }^{2}}\right) *{ ref}_{forest} - {ref}_{ground}}{{ref}_{snow} - {ref}_{ground}}$$

with ${t}_{\lambda }^{2}$ being the transmissivity layer (see Auxiliary datasets) and ${ref}_{forest},{ref}_{snow}$ and ${ref}_{ground}$ being generally applicable reflectance values for forest, snow and ground for AVHRR GAC data^18^.

The post-processing includes another scanning for unrealistic snow-covered pixels. A certainly snow-free pixel mask, ${px}_{snow-free}$, is applied to all data when the reflective part of channel 3b is used:

6 $${px}_{snow-free} \, if \, difference \, vis_{red}-ref3b<0.1 \, AND \, ref3b>0.2$$

The used ${ref}_{forest},{ref}_{snow}$ and ${ref}_{ground}$ in the SCAmod main algorithm are based on AVHRR GAC specific reflectance values from channel 1. We used 12 regions of interest (ROIs) for ${ref}_{forest}$ and ${ref}_{ground}$, respectively. The ROIs for ${ref}_{forest}$ are located in landcover classes ‘crop’, ‘sparse’, ‘grass’, ‘wetlands’ or ‘shrubs’ according to the ESA CCI Landcover information in the year 2000, while the ROIs for ${ref}_{ground}$ are located in areas where ‘mixed’, ‘needle trees’ or broadleaf trees’ were mapped. We used an investigation period of 5 years centring around the year 2000, i.e. 1998 to 2002. The monthly minimum value of channel 1 reflectance throughout the summer half-year (AMJJAS) was calculated per year and landcover class and subsequently averaged per class and study period as well as over all landcover classes. For ${ref}_{snow}$ the ROIs of ${ref}_{ground}$ were used, but an additional condition, namely $NDSI>0.7$ was implemented. In contrast to ${ref}_{forest}$ and ${ref}_{ground}$, the investigation period for ${ref}_{snow}$ covered winter and spring months, i.e. January to May. This analysis resulted in a ${ref}_{forest}$ value of 0.115, a ${ref}_{ground}$ value of 0.136 and a ${ref}_{snow}$ value of 0.558.

Lastly, for the time series analysis of this study, a temporal gap-filling after^19^ to mitigate the influence of clouds is applied. We combined a forward and backward (pixel value of the closest clear-sky observation in the forward and backward direction, respectively) gap-filling procedure over a maximum period of ± 3 days in both directions. No averaging or temporal weighing was applied, rather the closest possible information was used to fill missing information due to cloud obscureness.

### Snow cover metrics and trend analysis

To analyse the temporal distribution and occurrence of seasonal snow cover both the Snow Cover Area Percentage (SCA %) and the Snow Cover Duration (SCD) are frequently used measures^21,22^ and of broad interest. SCA % is defined as the ratio of snow covered pixels in a pre-defined reference area to all pixels within the reference area at a specific point in time. SCD is defined as the pixel-wise sum of snow days for one hydrological year. Moreover, we obtained early (SCD_es_, 1 September to 15 January) and late (SCD_lt_, 16 January to 31 August) season SCD after^26^. All SCA and SCD metrics are based on binary snow cover information, assuming a pixel being snow-covered if the fractional snow cover information was ≥ 50%. This choice is based on (i) a longstanding history of linking NDSI values of ≥ 0.4 with 50% snow coverage and more^66–68^, and (ii) to be in line with the NOAA CDR that uses the same threshold^23^.To investigate trends over the entire study period, standardised and non-standardised anomalies of SCA %, at varying spatial scales and temporal frequency were calculated, using the reference period 1982–2010. The reference period choice is based on the recommendations by the World Meteorological Organisation for remotely sensed datasets^69^. Trendlines and the associated Senn slope and Kendall p were obtained applying the non-parametric Mann Kendall test implemented in pyMannKendall^70^ with a confidence level of α equal to 0.05.

### Limitations in the AVHRR GAC dataset

The 37-year-long timeseries, based on seven AVHRR sensors onboard NOAA satellites, is intercalibrated and geolocated to ensure long-term data stability^16^. The individual satellites undergo drift effects throughout their life span^71^, leading to a change in local observation time and thus to a change in solar zenith angle. These effects can impact the consistency of the timeseries. However, this affects mainly the retrieval of parameters with a distinct diurnal cycle, while the cloud detection efficiency and the retrieval of snow cover are likely to be affected marginally. Furthermore, the retrieval of SSC is not affected by this effect, as we base our analysis on binary snow cover information and thus exclude slightly covered snow pixels, which may undergo a diurnal cycle. Yet, the use of binary snow information may lead to a slight underestimation of snow cover area at a local scale. Additionally, the spatial resolution of 0.05° may have a few limitations for the retrieval of snow cover, however an analysis based on in situ station data and high-resolution satellite data showed great overall accordance of detected snow cover extents^20^. Moreover, the long-term consistency of our snow cover dataset is proven by the direct comparison with MERRA2 and NOAA CDR (see Supplementary Fig. S1), showing the non-existing impact of orbit drift on the snow cover retrieval.

## Supplementary Information

Supplementary Information.

## Data Availability

The CCI + Snow project datasets, the basis for this study, are freely available through the ESA Open Data Portal: https://climate.esa.int/en/odp/#/project/snow.
