# Supplementary material for: Revealing four decades of snow cover dynamics in the Hindu Kush Himalaya
Source: Sci Rep. 2022 Aug 4;12:13443. doi: 10.1038/s41598-022-17575-4 (PMC9352756; doi:10.1038/s41598-022-17575-4)
Supplement: Supplementary file 1 — Supplementary Information. [file 41598_2022_17575_MOESM1_ESM.pdf]

Supplementary Materials for

Revealing four decades of snow cover dynamics in the Hindu Kush Himalaya

Naegeli, K.*, Franke, J., Neuhaus, C., Rietze, N., Stengel, M., Wu, X. and Wunderle, S.

*Corresponding author. Email: kathrin.naegeli@geo.uzh.ch

This PDF file includes:

Tables S1 to S5
Figs. S1 to S6

Table S1.

Topographic and morphologic characteristics of HKH and the individual basins. Elevation information are based on the void-filled SRTM digital elevation model. Please note that some basins are cut by the HKH outline and thus given parameters are only representative for the area captured in this study.

	**Area** (km^2)	**Elevation minimum** (m a.s.l)	**Elevation maximum** (m a.s.l)	**Elevation median** (m a.s.l)	**Permanent ice** (% of region)	**Water** (% of region)
HKH	4,193,178	0	7390	3705	1.3	0.9
Amu Darya	104,943	255	5694	2525	1.3	0.0
Brahmaputra	431,046	28	6526	4481	2.9	0.3
Ganges	246,202	63	7390	2118	4.2	0.1
Huang He	219,286	1764	5449	3734	0.1	0.7
Indus	584,721	75	6680	3055	3.2	0.4
Irrawaddy	198,675	0	4873	930	0.0	0.1
Mekong	133,958	386	5419	4207	0.2	0.2
Tibetan Plateau	792,147	2668	6180	4755	0.7	3.1
Northwest	216,188	258	4340	2255	0.0	0.0
Salween	214,960	285	5722	3612	0.7	0.2
South	125,879	0	2476	301	0.0	0.0
Southwest	200,034	32	3115	873	0.0	0.9
Tarim	139,275	4218	6386	5100	2.3	1.8
Yangtze	563,532	713	6044	4181	0.3	0.2

Table S2.

Monthly mean SCA % for the period 1982-2018 for entire HKH and all individual basins (numeric details of Figure 1c). Please note that some basins are cut by the HKH outline and thus given trend information are only representative for the area captured in this study.

	**Jan**	**Feb**	**Mar**	**Apr**	**May**	**Jun**	**Jul**	**Aug**	**Sep**	**Oct**	**Nov**	**Dec**
HKH median	14.9	14.7	11.0	7.5	3.7	1.2	0.4	0.3	0.6	3.8	6.5	8.2
HKH mean	16.1	15.0	11.3	7.7	4.1	1.5	0.5	0.4	0.9	4.7	7.5	10.1
HKH min	0.0	0.0	0.0	0.0	0.0	0.0	0.0	0.0	0.0	0.0	0.0	0.0
HKH max	43.0	40.2	27.9	19.7	12.2	7.6	2.1	1.9	6.1	17.7	23.1	35.1
Amu Darya	45.8	46.0	35.5	25.2	12.2	3.5	0.5	0.2	0.7	5.6	17.9	31.9
Brahmaputra	10.6	11.8	10.3	7.7	4.2	1.2	0.8	0.7	1.0	3.1	6.0	6.5
Ganges	10.0	12.2	10.4	5.6	1.5	0.4	0.4	0.4	0.5	1.8	2.7	4.6
Huang He	13.7	12.6	10.9	6.8	2.6	0.4	0.0	0.0	0.5	7.1	11.5	9.4
Indus	29.9	31.3	27.3	21.3	10.8	3.5	0.9	0.5	1.3	4.8	10.2	17.8
Irrawaddy	1.3	1.3	1.1	0.7	0.5	1.1	1.2	1.2	1.1	0.6	0.8	1.0
Mekong	10.7	9.3	8.2	6.5	3.0	1.1	0.3	0.3	0.8	6.7	9.7	8.3
Northwest HKH	45.8	46.9	24.8	5.2	0.2	0.0	0.0	0.0	0.0	0.2	5.7	24.3
Salween	11.4	9.4	8.4	8.0	4.9	1.9	0.8	0.7	1.1	7.0	10.8	9.2
South HKH	0.0	0.0	0.0	0.0	0.2	0.9	1.4	1.3	1.1	0.5	0.2	0.1
Southwest HKH	0.4	0.2	0.0	0.0	0.0	0.0	0.0	0.0	0.0	0.0	0.0	0.1
Tarim	21.0	14.8	9.1	7.0	7.3	4.6	0.7	0.5	3.3	10.8	10.0	12.8
Tibetan Plateau	14.3	9.5	4.6	3.2	2.9	1.2	0.2	0.2	1.1	6.1	7.0	8.9
Yangtze	9.7	8.2	7.5	5.8	3.0	0.8	0.1	0.1	0.7	6.8	9.5	8.5

Table S3.

Seasonal changes in SCA % for the period 1982-2018 for entire HKH (numeric details of Figure 3).

Year	Jan	Feb	Mar	Apr	May	Jun	Jul*	Aug*	Sep	Oct	Nov	Dec*
1982	−0.680	0.843	1.044	1.163	0.678	0.427	0.807	0.414	−1.025	0.002	0.999	1.354
1983	1.882	0.860	1.056	1.266	1.669	2.418	2.972	0.002	−0.374	1.320	−0.718	−0.490
1984	−0.818	−0.961	−1.708	−1.860	−1.169	−0.871	−0.750	−0.461	−0.526	−1.025	−0.968	−1.049
1985	0.148	−1.270	−1.452	−0.642	−0.547	0.302	0.155	−0.722	1.157	1.129	1.399	1.147
1986	−0.099	−0.015	−0.851	0.208	1.151	0.310	0.134	−0.465	0.413	0.694	−0.288	1.012
1987	−0.517	−1.061	−1.004	−0.035	1.448	0.142	0.692	0.583	0.054	0.526	−0.633	0.588
1988	0.252	−0.468	0.710	0.071	−0.326	−0.491	−0.952	−0.405	0.881	−0.965	−1.169	0.170
1989	2.271	0.913	0.121	1.194	1.364	1.808	0.548	1.415	−0.084	−0.152	0.627	1.282
1990	0.126	0.997	1.525	1.182	0.848	−0.591	−0.010	−0.104	0.494	−0.012	−1.068	−0.537
1991	1.528	−0.588	0.689	0.144	−0.390	−0.133	−0.008	0.502	−0.577	−1.418	−0.874	0.111
1992	0.003	0.531	−0.420	−0.403	−0.463	0.250	0.307	−0.362	−0.351	−0.396	−1.087	−0.617
1993	1.268	−0.829	0.348	−0.321	−0.845	−0.767	0.083	−0.197	0.731	−0.728	−1.137	−1.228
1994	−0.497	0.446	−0.223	0.348	−0.480	−0.678	−0.555	0.195	−0.577	−2.199	-	-
1995	-	0.976	1.051	1.461	−0.411	−0.505	0.796	0.069	0.241	−0.725	0.909	1.046
1996	1.242	0.064	0.358	0.388	−0.186	1.048	−0.705	0.030	−0.086	0.298	0.356	−1.060
1997	−0.665	0.573	0.074	1.076	−0.128	−0.126	−0.487	0.114	0.039	1.795	1.848	2.582
1998	1.105	1.040	1.443	0.834	−0.869	−0.935	−0.597	−0.367	−0.714	1.125	0.108	−0.682
1999	−0.530	−1.120	−0.429	−1.514	−1.013	−0.627	−0.675	−0.296	−0.897	0.919	0.017	−0.813
2000	−1.045	0.163	−0.444	−1.172	−0.772	−1.812	−1.710	−1.028	0.024	−1.189	−1.486	−1.361
2001	−1.780	−2.185	−1.278	0.742	1.322	1.749	1.443	2.270	1.126	−0.933	−0.205	0.387
2002	0.603	0.513	1.629	0.991	2.255	1.408	1.285	3.710	3.900	1.147	1.182	−0.352
2003	−1.044	0.521	0.820	0.440	0.802	−0.505	0.003	−0.759	−0.891	−1.432	−0.813	−0.407
2004	−0.420	−0.557	−2.037	−1.972	−1.198	−0.799	−1.265	−0.678	−1.151	0.670	−0.107	−0.546
2005	0.147	0.277	0.005	0.458	0.245	0.404	−1.172	−1.224	−0.712	0.345	0.507	−0.935
2006	−1.137	−1.158	0.069	−0.139	−0.632	−1.051	−0.376	−0.604	−0.710	−0.113	0.845	1.421
2007	−0.879	0.287	1.015	−1.284	−1.429	−0.309	−0.861	−0.511	−0.387	−0.618	−0.837	−0.093
2008	0.476	2.722	−0.714	−1.138	−1.166	−1.318	−0.947	−0.539	1.002	0.943	1.886	−0.074
2009	0.017	−0.759	−0.556	−0.431	0.491	0.251	0.413	−0.471	−0.607	1.107	1.301	0.412
2010	−0.955	−0.758	−0.841	−1.055	−0.246	1.002	1.434	−0.111	−0.392	−0.117	−0.591	−1.267
2011	−0.010	0.149	0.475	0.703	−0.208	0.013	−0.450	0.283	−0.530	−0.273	0.610	−0.914
2012	1.249	0.905	1.131	0.304	0.877	−0.121	0.132	−0.198	−0.921	−0.527	−0.728	−0.053
2013	−0.212	0.871	−0.193	−0.475	1.011	−0.220	−0.214	−0.262	−0.245	0.239	1.848	−0.275
2014	−0.375	0.304	0.094	0.136	−0.569	−0.277	−0.684	−0.148	0.565	0.114	−0.458	−0.099
2015	0.894	−0.824	0.205	0.831	−0.300	−0.241	−1.006	−0.698	−0.780	−1.316	−0.732	−0.495
2016	−0.913	−1.412	−0.969	−1.098	−0.647	−1.209	−1.369	−0.978	−0.118	−1.045	−1.516	−1.261
2017	−0.594	−0.314	0.163	−0.701	−0.982	−0.406	−0.119	−1.283	−0.890	−1.263	−0.937	−1.194
2018	−2.144	−1.998	−0.806	−1.020	−1.391	−1.768	−1.931	−1.173	0.058	−0.243	−0.567	−1.029
Senn slope	−0.024	−0.013	−0.009	−0.027	−0.024	−0.026	−0.036	−0.022	−0.011	−0.015	0.001	−0.031
Kendall p	0.089	0.425	0.628	0.097	0.082	0.058	0.012	0.019	0.224	0.266	0.989	0.032

*Months marked with asterisk exhibit a significant trend over the study period. For cells marked with ‘-’ no data available.

Table S4.

Spatio-temporal variability in snow cover extent for the period 1982-2018 for entire HKH and all individual basins (numeric details of Figure 4). Some basins are cut by the HKH outline and thus given trend information are only representative for the area captured in this study.

Year	HKH	AD	BP	GA	HH	IN	IW	MK	NW	SA	S	SW	TA	TP	YA
1982	1.41	0.52	1.30	1.48	2.89	2.03	0.05	0.12	0.81	6.52	1.01	0.20	0.07	3.98	0.23
1983	1.83	−1.00	0.79	1.18	4.26	3.11	0.35	1.68	1.77	2.90	0.86	0.16	−0.02	5.45	1.78
1984	−2.56	−3.02	−2.39	−1.77	−2.88	−3.44	−0.15	−2.29	−3.58	−1.27	−2.61	−0.21	−0.01	−5.23	-2.50
1985	0.30	−1.14	−0.61	−0.09	−0.23	−1.04	−0.29	−0.04	1.40	−1.73	−0.32	−0.12	−0.02	2.95	2.32
1986	0.49	0.56	0.28	1.02	−1.61	0.83	−0.11	−0.64	0.23	2.77	0.30	−0.17	0.05	1.84	0.80
1987	−0.39	−1.01	0.16	1.15	−2.01	1.79	−0.18	−0.69	−0.35	−5.63	−1.46	−0.14	−0.05	−0.93	-0.61
1988	−0.24	−1.82	−0.35	−0.01	−1.52	1.27	0.04	−1.23	0.06	−0.12	−0.76	−0.18	−0.06	0.73	-1.53
1989	2.58	4.51	1.46	1.02	5.99	3.05	0.35	2.55	3.36	5.54	2.35	0.25	−0.01	1.63	2.03
1990	0.59	0.32	0.12	−0.49	2.24	0.16	0.08	2.30	−0.24	3.90	2.07	0.11	0.06	−1.12	1.12
1991	0.20	2.09	−0.22	0.61	−0.39	2.22	0.03	1.15	−1.44	4.02	0.25	0.01	0.03	−1.43	-0.69
1992	−0.66	1.74	−0.58	−0.84	−1.64	0.57	−0.05	−0.92	−2.33	2.96	−1.27	−0.03	−0.04	−2.42	-0.79
1993	−0.75	0.65	−1.34	−0.34	2.11	−1.01	−0.08	−1.22	−1.26	0.28	−2.23	−0.18	0.01	−2.56	-0.75
1994*	−1.22	−0.61	−0.27	−0.04	−1.09	1.45	0.14	−2.16	−3.20	−3.14	−2.49	0.00	0.03	−1.93	-2.05
1995*	0.66	−0.05	0.68	1.76	3.33	−0.87	0.04	2.92	1.24	−2.65	1.51	0.28	−0.05	−2.65	1.86
1996	0.48	2.21	1.20	1.66	0.05	0.93	−0.28	2.40	−0.19	−0.93	1.05	−0.02	0.04	−1.26	0.69
1997	2.14	−1.99	1.59	1.02	4.57	0.49	−0.18	2.51	3.93	1.19	3.86	−0.14	0.08	4.68	3.03
1998	1.12	−2.91	0.79	1.11	−1.06	2.22	−0.32	0.88	1.98	0.01	2.12	−0.22	−0.02	3.67	1.30
1999	−1.32	−1.49	−2.22	−1.62	−2.16	−2.03	−0.44	−1.21	−0.69	−3.69	−1.29	−0.07	−0.03	−0.52	-0.65
2000	−2.14	−5.23	−1.45	−1.40	−3.02	−4.28	−0.32	−1.36	−1.83	−4.83	−1.21	−0.21	0.01	−3.94	-1.02
2001	−1.34	−0.90	0.03	−0.67	−1.93	−2.56	0.92	−1.11	−1.67	−5.89	−0.41	0.82	−0.04	−0.75	-1.21
2002	2.10	3.71	3.23	3.45	−0.56	3.17	1.64	0.85	3.51	−1.56	1.95	0.53	−0.04	4.96	0.55
2003	−0.61	3.30	1.48	1.16	−3.21	0.23	0.18	−1.94	−1.24	−0.21	−1.03	0.11	−0.02	−2.73	-2.38
2004	−1.60	−0.54	−0.73	−2.20	−2.52	−3.09	−0.37	−2.06	−0.86	−3.04	−0.69	−0.16	0.01	−3.97	-1.71
2005	0.04	1.50	0.57	0.08	0.87	−0.15	−0.29	0.65	−0.82	1.35	0.59	−0.28	0.05	−0.70	-0.03
2006	−0.15	1.35	−0.97	−1.66	−1.47	−0.37	−0.27	0.19	0.61	0.60	−0.13	−0.01	−0.03	1.57	-0.43
2007	−0.81	−2.90	−0.36	−0.67	−1.69	−3.10	0.02	−2.05	0.21	2.04	−0.97	−0.11	−0.05	−1.29	-0.87
2008	1.23	1.29	0.59	−1.28	5.80	−0.83	−0.01	3.19	1.38	3.04	1.98	−0.22	0.12	1.72	2.37
2009	0.39	3.47	−1.03	−1.77	0.76	1.01	−0.24	0.01	0.32	1.82	−0.98	−0.08	−0.01	2.93	0.88
2010	−1.76	−2.61	−1.77	−1.83	−3.86	−1.77	−0.24	−2.47	−1.10	−4.26	−2.05	0.09	−0.05	−2.68	-1.74
2011	−0.02	−0.59	−0.04	−0.21	−0.44	0.26	0.11	0.97	−0.09	−1.29	0.78	0.12	−0.01	−0.35	0.14
2012	1.00	5.09	0.15	−0.32	4.13	−0.32	−0.13	1.88	0.99	4.17	0.39	0.04	0.07	1.99	0.98
2013	0.64	1.95	0.77	1.43	−0.63	1.44	−0.35	1.16	0.69	−0.64	0.31	0.21	−0.05	0.99	0.68
2014	−0.19	0.83	−0.81	1.16	−0.73	0.64	−0.27	−1.32	0.63	0.37	−1.68	−0.01	−0.02	−1.44	-1.58
2015	−0.43	1.83	−0.21	1.81	−2.44	1.63	−0.53	−1.27	0.17	−2.50	−2.27	−0.02	−0.05	−1.54	-2.43
2016	−2.61	−3.39	−2.10	−2.70	−2.88	−4.48	−0.24	−2.41	−2.44	−4.58	−2.87	−0.15	−0.06	−4.07	-2.00
2017	−1.64	−2.18	−1.94	−1.33	−0.73	−1.75	−0.44	−2.41	−1.92	−1.72	−2.54	−0.30	0.11	−3.21	-1.66
2018	−2.90	−5.87	−1.51	−2.18	−1.98	−4.97	−0.44	−1.96	−3.13	−8.42	−2.50	−0.32	−0.04	−4.63	-1.63
Senn slope	−0.049	0.003	−0.033	−0.051	−0.043	−0.079	−0.010	−0.044	−0.110	−0.059	−0.003	−0.001	−0.086	−0.030	-0.046
Kendall p	0.040	0.969	0.082	0.065	0.154	0.035	0.008	0.092	0.073	0.065	0.425	0.289	0.048	0.440	0.055

*Please not the gap in data availability between November ’94 and end of January ’95 might influence the two respective years.

Table S5.

Channel configuration of AVHRR-2 and AVHRR-3 as well as prime satellites on-board NOAA platforms and used for the presented time series.

channel	AVHRR-2 (µm)	AVHRR-3 (µm)
1	0.58-0.68	0.58-0.68
2	0.73-1.10	0.73-1.0
3A	-	1.59-1.63
3B	3.55-3.93	3.55-3.93
4	10.50-11.50	10.30-11.30
5	ch4 repeated	11.50-12.50

platform	NOAA-07			NOAA-09			NOAA-11							NOAA-14						NOAA-16				NOAA-18				NOAA-19									
year	´82	´83	´84	´85	´86	´87	´88	´89	´90	´91	´92	´93	´94	´95	´96	´97	´98	´99	´00	´01	´02	´03	´04	´05	´06	´07	´08	´09	'10	'11	'12	'13	'14	'15	'16	'17	'18

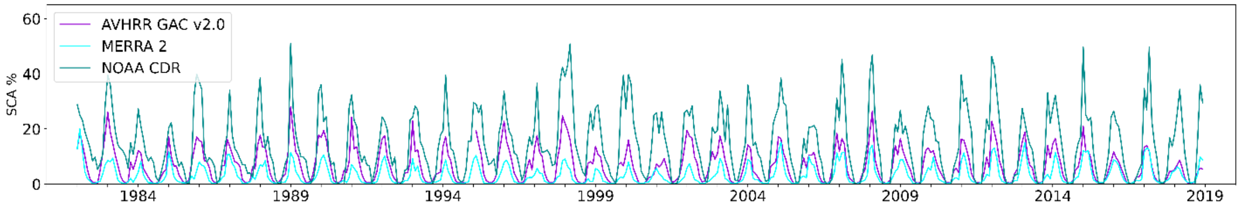

Fig. S1. Proof of temporal consistency of our AVHRR GAC SSC product in comparison with MERRA2 and NOAA CDR snow cover products. Due to differing datasets and methodologies, the AVHRR GAC SSC product ranks between the other datasets in absolute values and is consistent over time. For details about the individual datasets, their underlying data and methodologies, we refer to the existing literature.

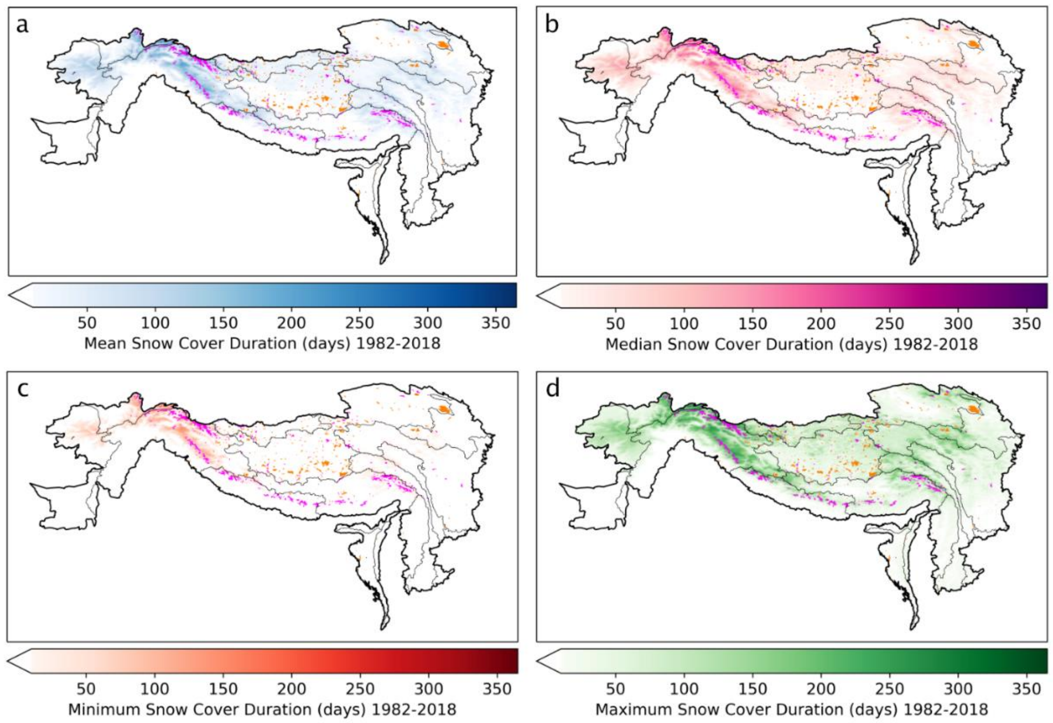

Fig. S2. Annual spatial Snow Cover Duration (SCD) metrics over the entire timeseries 1982-2018: a, Mean, b, Median, c, Minimum and d, Maximum. Permanent ice is displayed in pink, masked water bodies in orange.

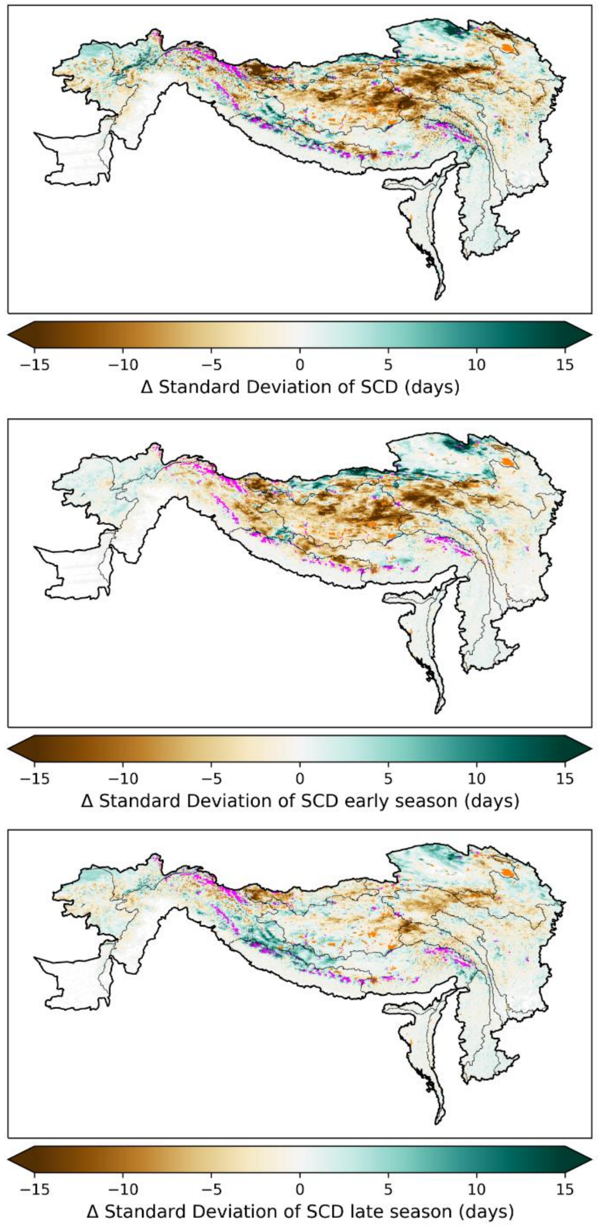

Fig. S3. Long-term Seasonal Snow Cover variability. Differences in mean standard deviation between the periods 2000 to 2018 and 1982 to 2000 for mean snow cover duration (top), mean early season snow cover duration (middle), and mean later season snow cover duration (bottom). Early season is defined as between 1 September and 15 January of a given hydrological year, while late season is defined as between 16 January and 31 August. Permanent ice is displayed in pink, masked water bodies in orange. Geolocation and scale according to figure 1.

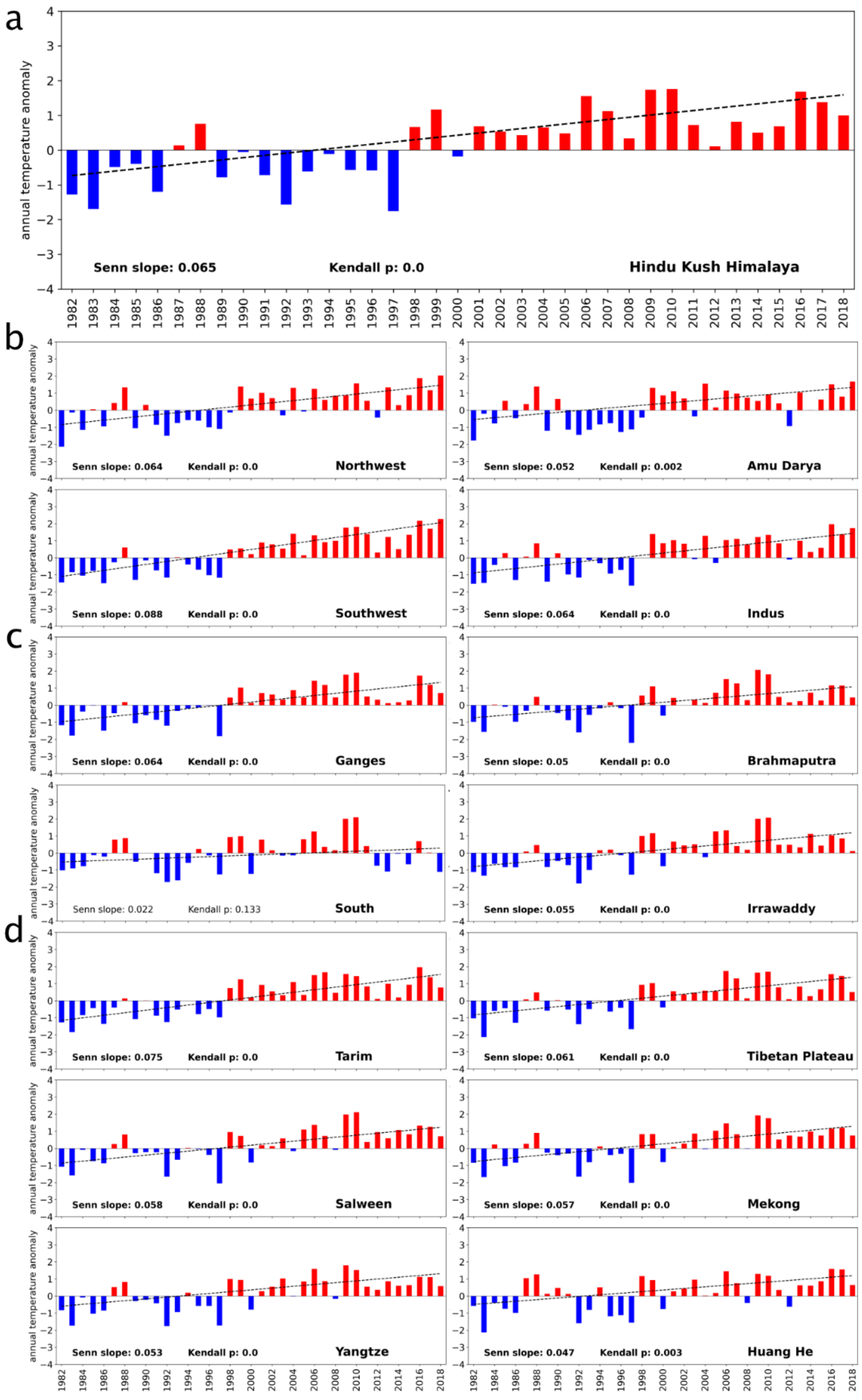

Fig. S4. Annual temperature anomalies based on CRU TS 4.0 data for the entire HKH region (panel a), and one of the 14 basins; arranged in greater regions west (panel b), central (panel c) and east (panel d), with respect to the 1982-2010 reference period. Dashed lines denote the MK trendline and statistics (Senn slope and Kendall p) are given at the bottom of each graph.

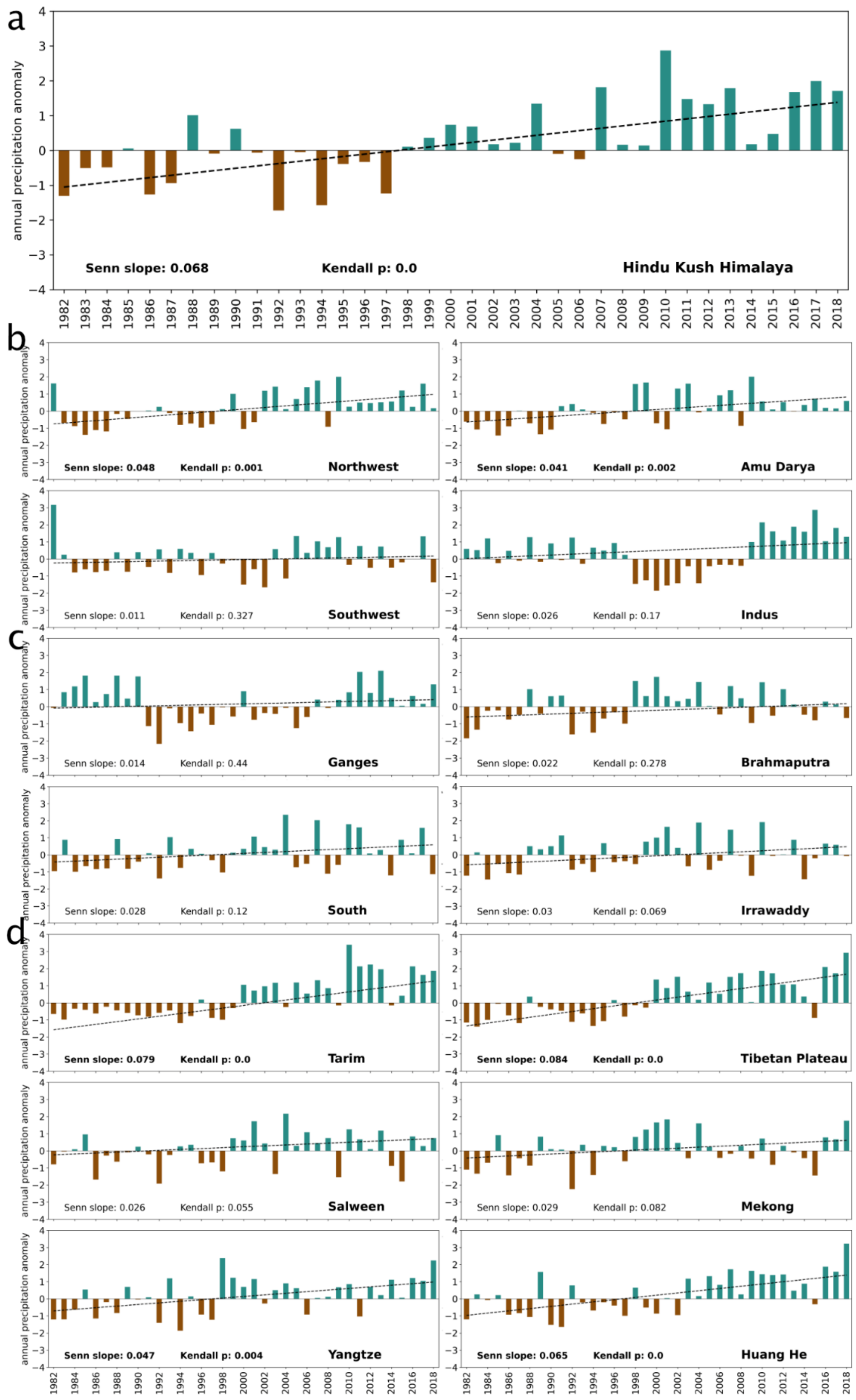

Fig. S5. Annual precipitation anomalies based on CHIRPS data for the entire HKH region (panel a), and one of the 14 basins; arranged in greater regions west (panel b), central (panel c) and east (panel d), with respect to the 1982-2010 reference period. Dashed lines denote the MK trendline and statistics (Senn slope and Kendall p) are given at the bottom of each graph.

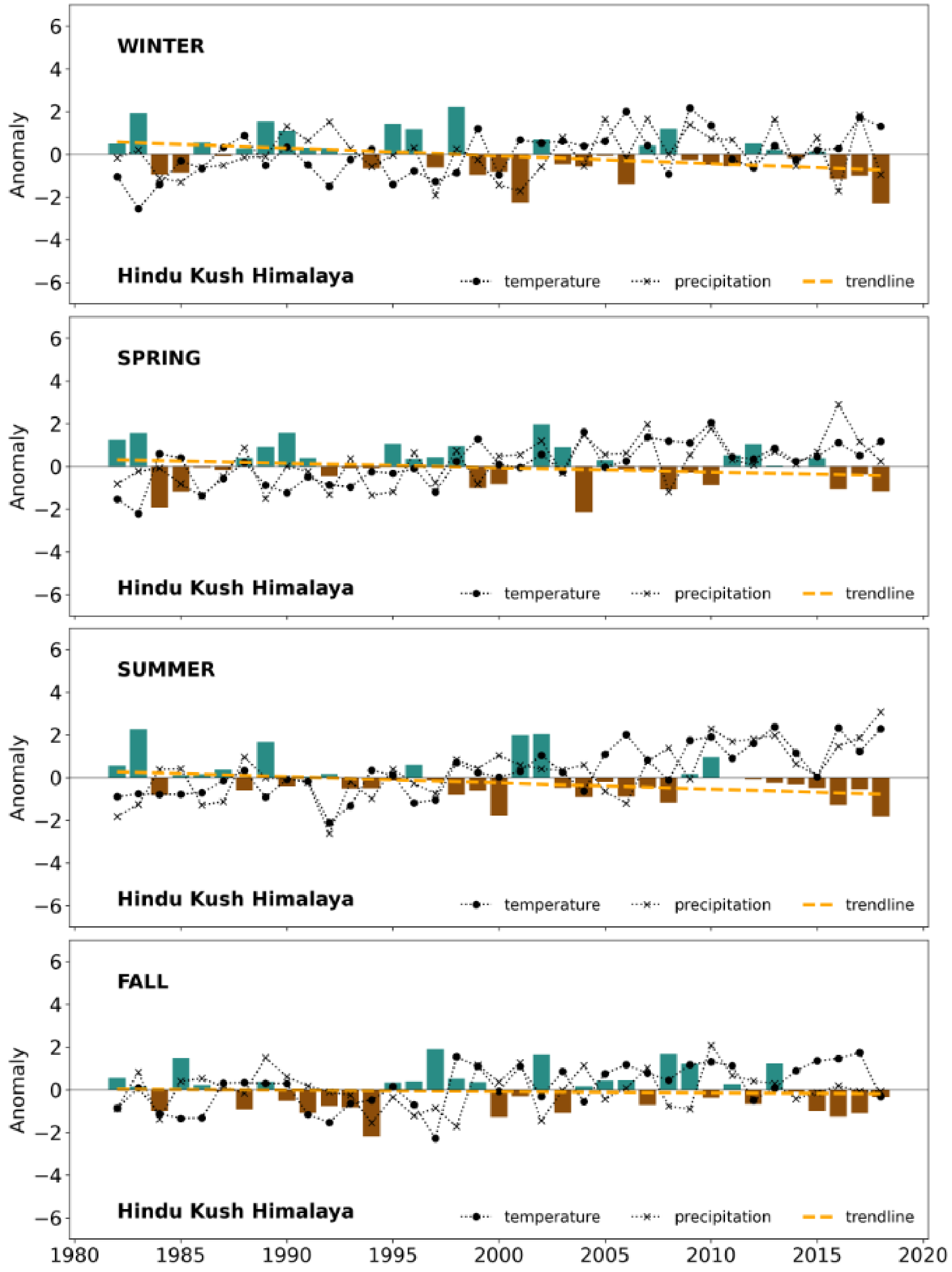

Fig. S6. Snow cover seasonality (bars, standardised seasonal SCA% anomalies) for entire HKH and its complex interplay with air temperature (dots, standardised annual anomalies in °C) and precipitation (crosses, standardised annual anomalies in mm). MK trendline of seasonal anomalies are plotted as yellow, dashed lines.
